# Supplementary material for: Unsupervised learning reveals interpretable latent representations for translucency perception
Source: PLoS Comput Biol. 2023 Feb 8;19(2):e1010878. doi: 10.1371/journal.pcbi.1010878 (PMC9942964; doi:10.1371/journal.pcbi.1010878)
Supplement: S1 Text — (PDF) [file pcbi.1010878.s001.pdf]

Supporting Information (S1 Text)

Experiment 1: Real-versus-generated discrimination

To further illustrate the results of Fig 2D in the main paper, S1 Fig shows examples of real-versus-generated judgment agreed by the majority of observers (at least 50% of observers).

Experiment 2: Material attribute rating

S2 Fig shows the illustrations of the light transport process for opaque and translucent objects that we presented to the observers in the experiment instruction.

Experiment 3: Perceptual evaluation of emerged scene attributes

Supplementary S1 Table summarizes the Bayesian multilevel multinomial logistic regression model results. We set the baselines of the variables in the following way: "lighting" for the scene attribute, "later-layer manipulation" for the layer-manipulation method, and "opaque-opaque (OO)" for the type of source-target pair.

The most salient output from the model is the mean of the posterior distribution for early-layer manipulation. The relative risk ratio of selecting "shape" in comparison to selecting "lighting" when the layer-manipulation method switches from later-layer manipulation to early-layer manipulation has an estimated mean posterior of 2644.90 (95% Highest Density Interval (HDI), [1106.94, 6783.08]). We could make the decision of whether to accept or reject the null value of a parameter based on the relation between HDI and region of practical equivalence (ROPE). ROPE is set for each parameter with a 0.1 range around 0 using the unexponentiated model. If none of the 95% HDI of the parameter distribution falls into the ROPE, we can reject the null value. If the 95% HDI of the parameter distribution completely falls into the ROPE, we accept the null as a credible value. Otherwise, the decision remains undecided. Since zero percent of the 95% HDI of the parameter distribution for early-layer manipulation falls inside the ROPE, we can reject the null value. Therefore, early-layer manipulation is a credible parameter, and it increases the probability that the observers choose "shape/orientation" as the most prominent change in the image sequence, regardless of the source-target pair of the materials. Secondly, the relative risk ratio of selecting "material" in comparison to selecting "lighting" when the layer-manipulation method switches from later-layer manipulation to middle-layer manipulation has an estimated mean posterior of 2.11 (95% HDI, [1.58, 2.80]). Middle-layer manipulation is also a credible parameter (Inside.ROPE = 0), and it increases the probability for the observers to select "material" as the most prominent visual attribute changed as compared to selecting "lighting". It is also important to note that the middle-layer manipulation applied on opaque-translucent pairs increases the probability that observers choose "material" as the most prominent visual attribute being changed (Mean Est = 11.61, 95% HDI, [7.34, 18.39], Inside.ROPE = 0). Lastly, later-layer manipulation is most likely to lead to the change in "color" across all source-target pair conditions.

S3 Fig illustrates the conditional effect of layer-manipulation on the prediction of the most prominent scene attribute chosen by the observers. For the early-layer manipulation, the estimated probability of selecting "shape" is close to 1 across all three types of source-target pair. For the later-layer manipulation, the estimated probability of selecting "color" is approximately

77.4% (95% HDI, [71.4%, 82.5%]) for OT pairs, 68.7% (95% HDI, [61.4%, 75.1%]) for OO pairs, and 80.0% (95% HDI, [73.9%, 85.0%]) for TT pairs. For the middle-layer manipulation, the estimated probability of selecting "material" is 77.9% (95% HDI, [69.5%, 84.5%]) for the OT pair, which is higher than that of the OO or TT condition.

Visualizing the effect of layer manipulation based on learned decision boundary of milky-versus-glycerin soap classification

As an extension of Fig 5D in the main paper, we show the image manipulation results based on the learned SVM decision boundary for each of the 18 layers of $W+$ latent space. S4 and S5 Fig demonstrate the manipulation along the positive and negative direction of the normal of the learned decision boundary.

S2 Table shows the correlation coefficients between each layer's SVM model prediction and the material attribute ratings obtained from Experiment 2.

Independent Component Analysis (ICA) for the intermediate generative representation

We used FastICA from *scikit-learn* for the independent component analysis of image patches extracted from the intermediate results of the generative process our trained generator. For the intermediate generative result from 64 $\times$ 64 tRGB layer, we conducted the analysis using 64 and 100 components, and found that similar sets of sparse features (i.e., middle-layer ICA kernels) were extracted. More details can be found in the Results section of the main paper.

We also conducted a similar control analysis based on the intermediate generative result from the early-layers. For the 3160 "high" translucent images used to obtain the middle-layer ICA kernels, we extracted their corresponding intermediate generative results from 16 $\times$ 16 tRGB layer (S7A Fig). Since a great number of the 16 $\times$ 16 generative results were generated from the same early-layer latent vectors, we randomly sampled 1000 of them to reduce the redundancy. For each image, we first resized it to 512 pixels $\times$ 512 pixels resolution, and then sampled 10 image patches of 96 pixels $\times$ 96 pixels from random locations. We then applied FastICA on the 1000 $\times$ 10 image patches to learn 64 basis functions (i.e., early-layer ICA kernels) (S7B Fig). The effect of convolving these 96 $\times$ 96 kernels with a real photograph of translucent soap is shown in S7C Fig. Compared with the information extracted from middle-layer kernels, the early-layer chromatic kernels detect coarser information of the edges, and cannot capture the fine spatial color variations that are indicative of the "glowing" effect of translucent materials.

Image generation with DCGAN

We used the DCGAN proposed by Radford et al. (2015) to train to generate 64 pixels $\times$ 64 pixels images of soaps. S8 Fig shows the examples of real photos from the training dataset and the DCGAN-generated results.

Computing image embeddings with dimensionality reduction methods

We use the embedding obtained from dimensionality reduction methods, t-SNE and MDS, to compute the SVM model prediction values (see Methods). S9 Fig shows the model prediction values resulted from the t-SNE embedding at various perplexity levels (5, 15, and 25), and from the MDS embedding. None of these embeddings show statistically significant correlation with human perceptual ratings of the 150 TAG-generated images in Experiment 2.
