# Supplementary figures and images for: Unsupervised learning reveals interpretable latent representations for translucency perception
Source: PLoS Comput Biol. 2023 Feb 8;19(2):e1010878. doi: 10.1371/journal.pcbi.1010878 (PMC9942964; doi:10.1371/journal.pcbi.1010878)

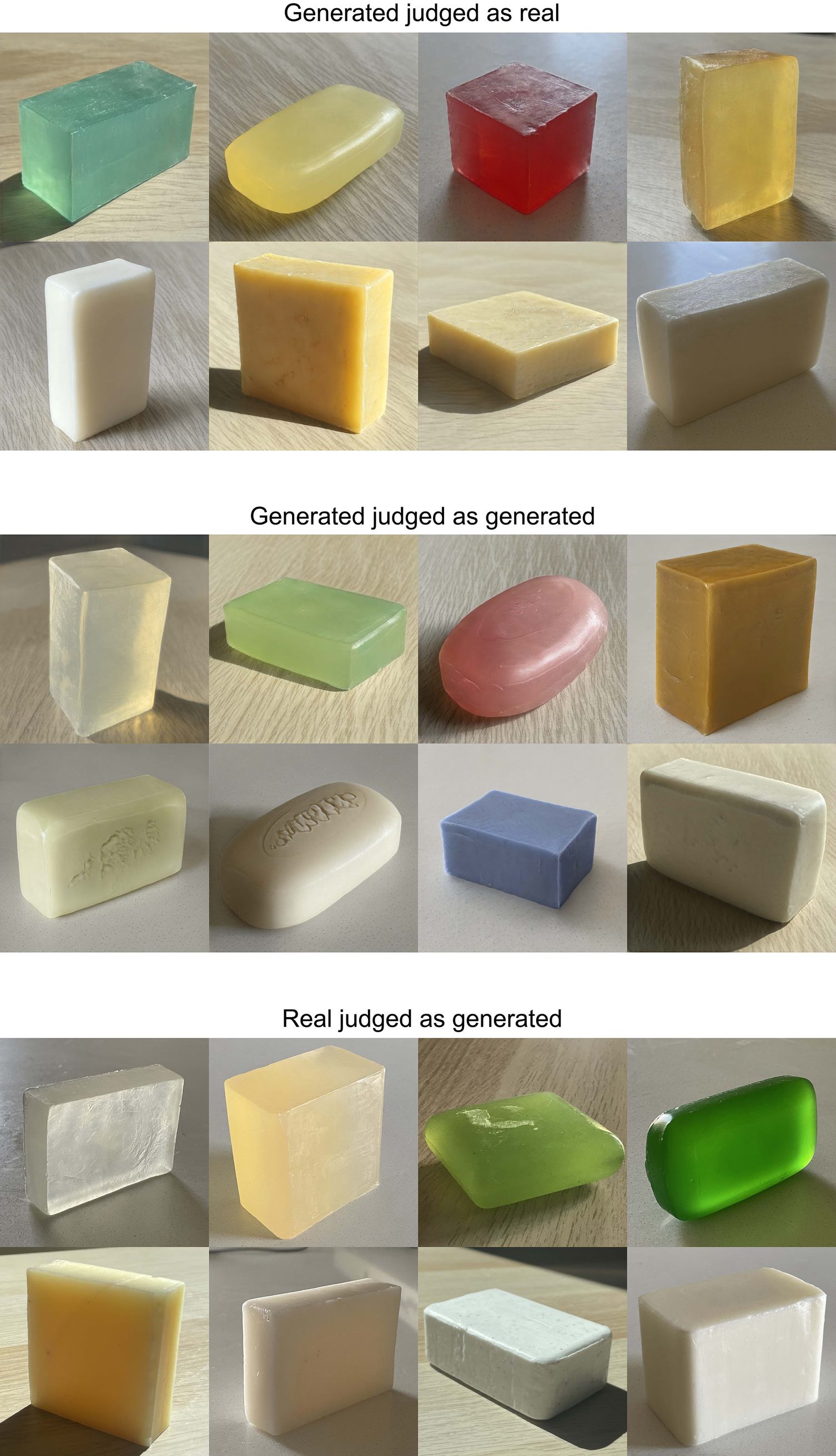

Supplement: S1 Fig — Each image is resized for display. (TIF) [file pcbi.1010878.s002.tif]

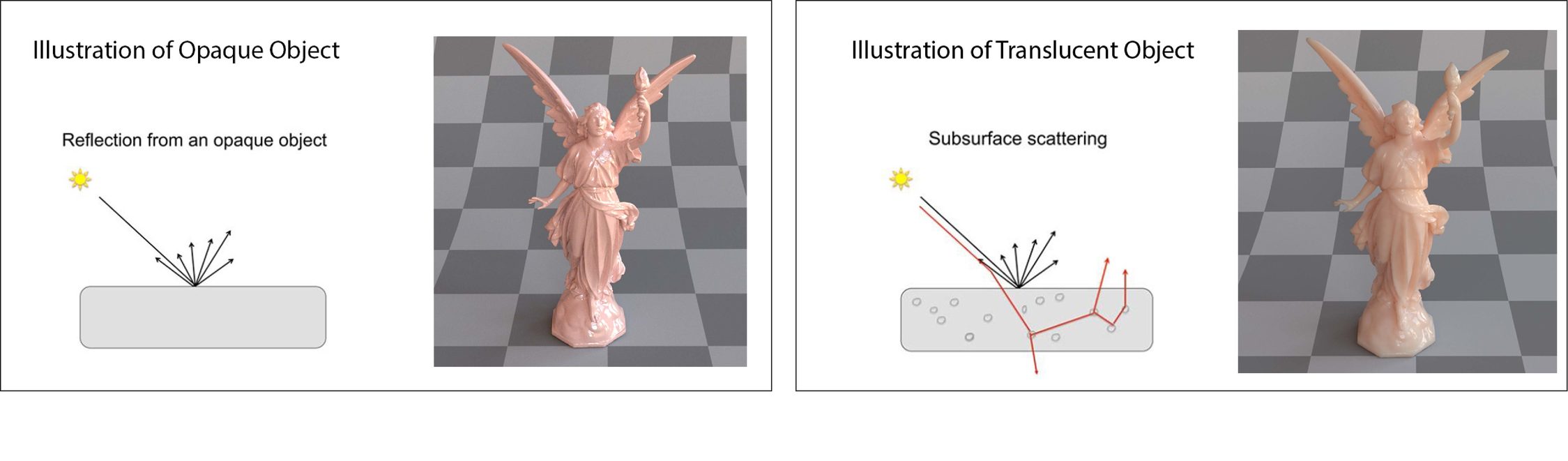

Supplement: S2 Fig — Left: light transport process for an opaque object. Right: subsurface scattering for a translucent object. (TIF) [file pcbi.1010878.s003.tif]

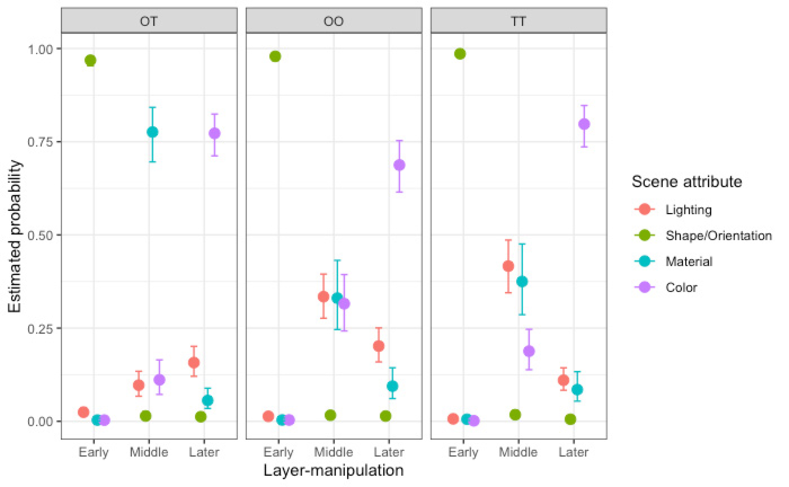

Supplement: S3 Fig — The x-axis is the layer-manipulation method, and the y-axis is the estimated probability that a certain scene attribute is selected as the most prominent attribute that has been changed in an image sequence. The error bar indicates the upper and lower bounds of the estimation at the confidence level of 95%. The panels show the predicted results for three source-target pair conditions: opaque-translucent (OT), opaque-opaque (OO), and translucent-translucent (TT). (TIF) [file pcbi.1010878.s004.tif]

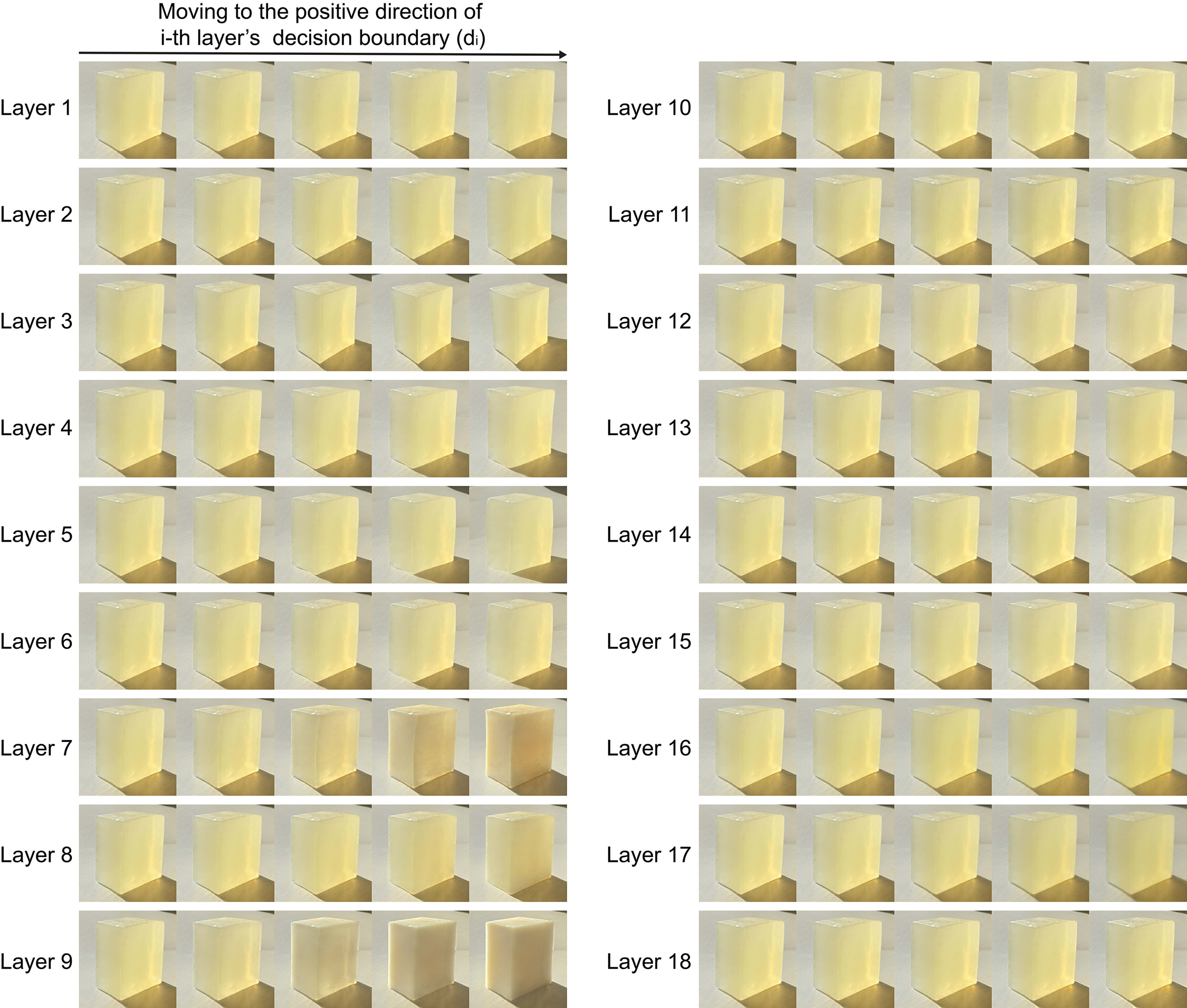

Supplement: S4 Fig — The displacement on the middle-layers (layers 7 to 9) can mainly affect the translucent appearance. (TIF) [file pcbi.1010878.s005.tif]

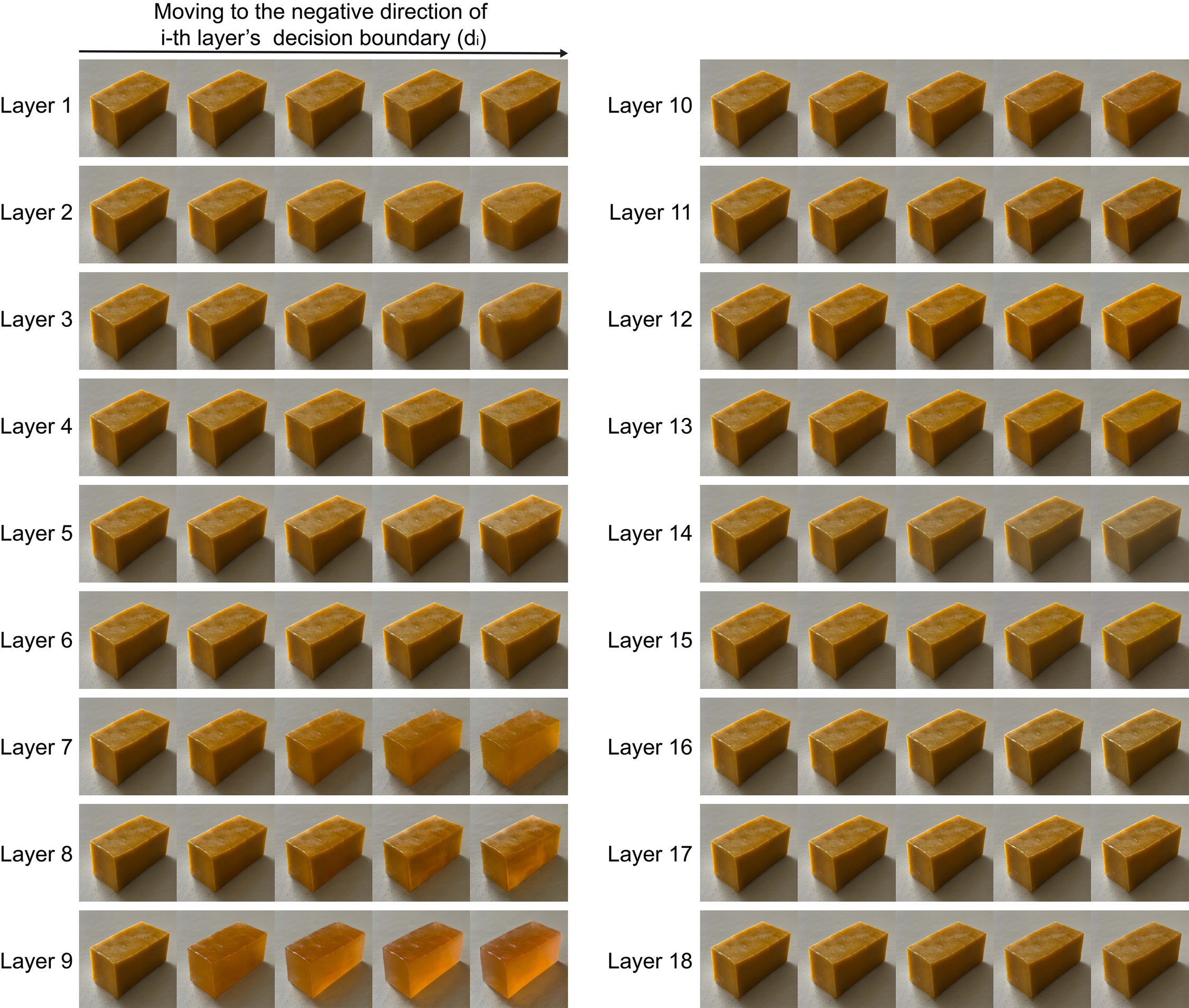

Supplement: S5 Fig — The displacement on the middle-layers (layers 7 to 9) can mainly affect the translucent appearance. (TIF) [file pcbi.1010878.s006.tif]

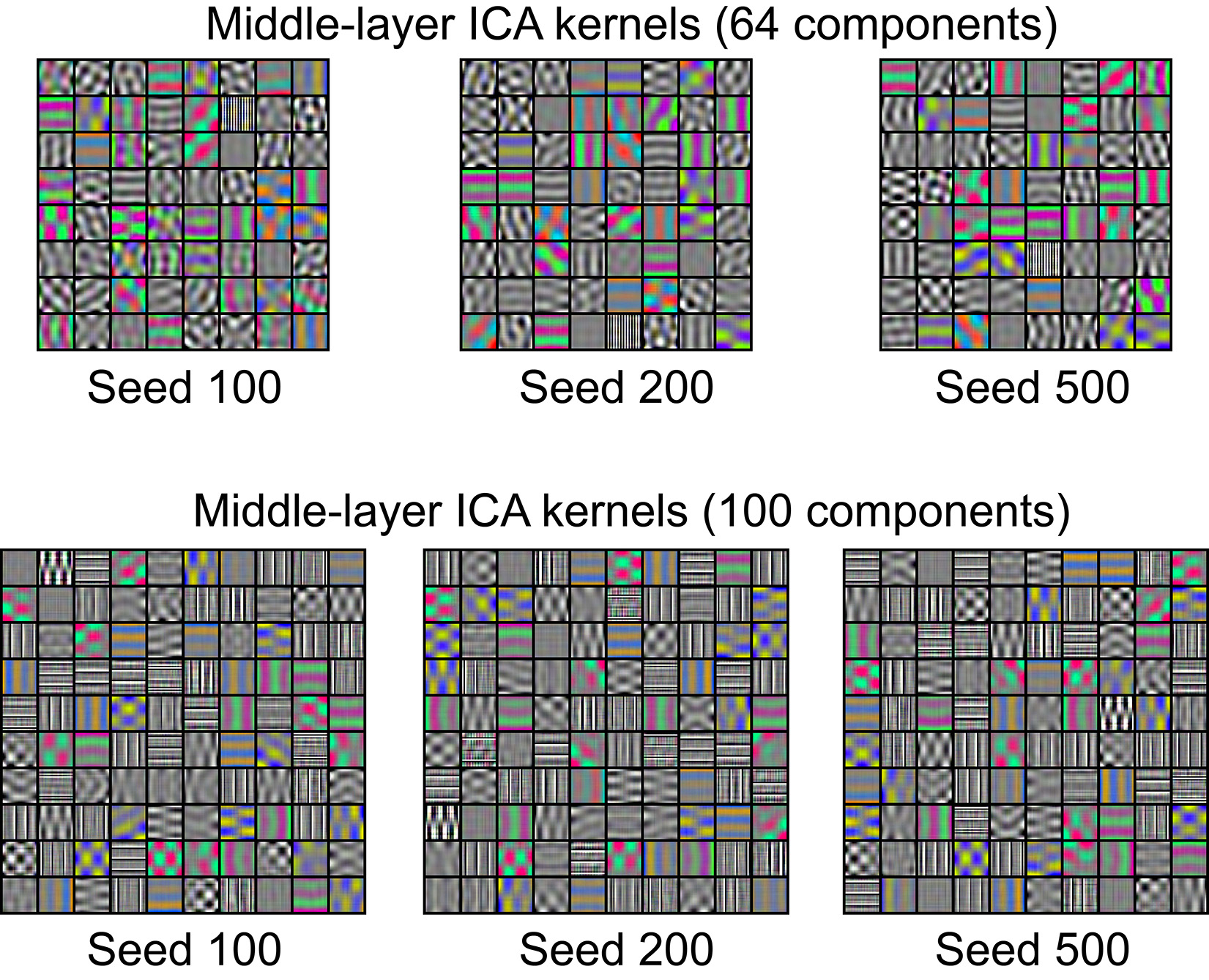

Supplement: S6 Fig — Top and bottom rows show the FastICA results of using 64 and 100 components respectively. Within each row, each panel shows the kernels learned from a different random sampling of the image patches. The kernels are 24 × 24 and are resized for display. (TIF) [file pcbi.1010878.s007.tif]

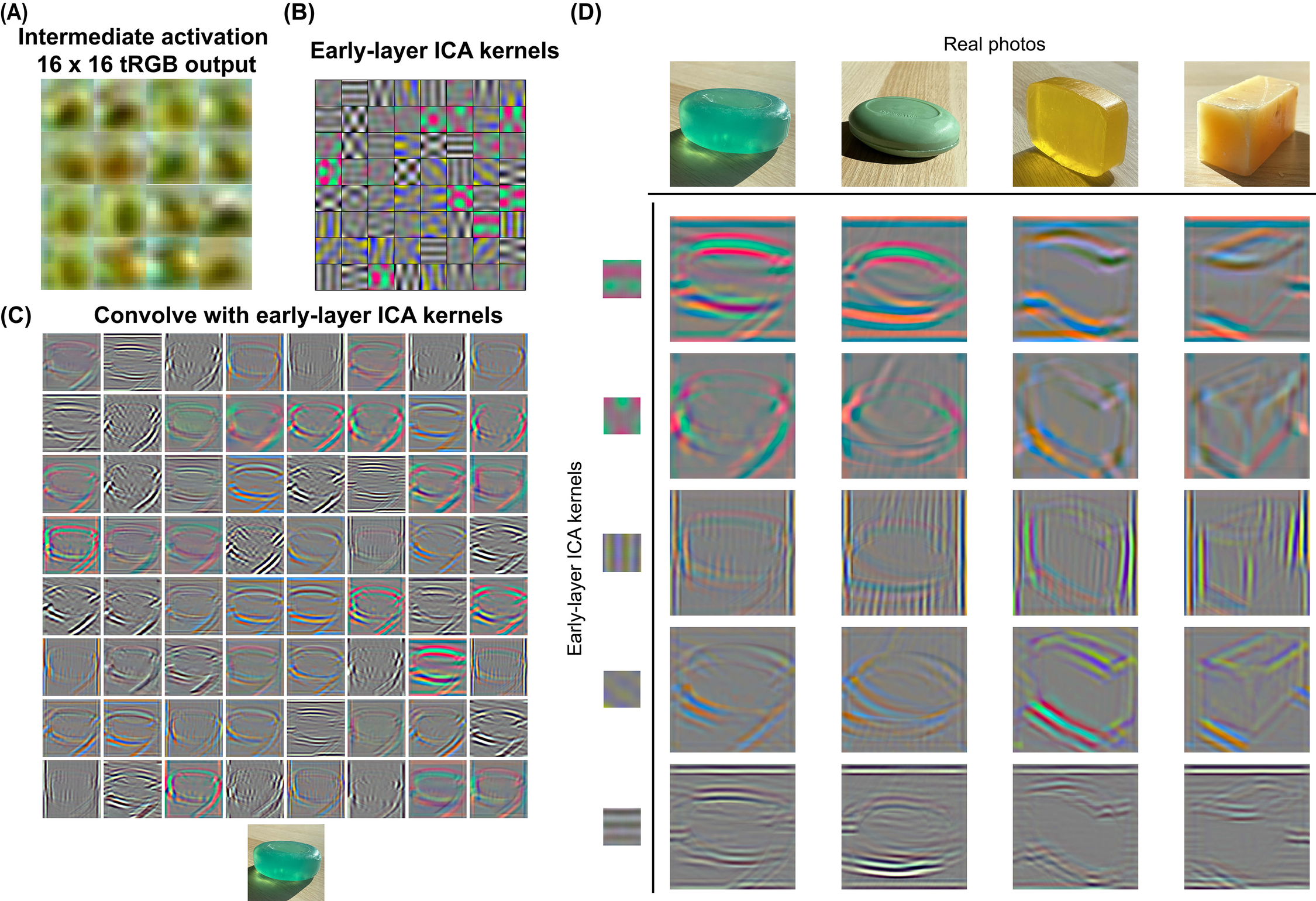

Supplement: S7 Fig — (A) The intermediate generated results (tRGB layer output at 16 pixels × 16 pixels resolution) of the images from the high-translucency dataset. The images are resized for display. (B) Early-layer ICA kernels obtained by training a system of 64 basis functions on 96 pixels × 96 pixels image patches extracted from images in (A). The kernels are of size 96 × 96. (C) Visualization of applying three-dimensional convolution of the individual early-layer ICA kernels in (B) on a real photograph of translucent soap. (D) The resulting filtered images of four different soaps with selected chromatic and achromatic kernels. (TIF) [file pcbi.1010878.s008.tif]

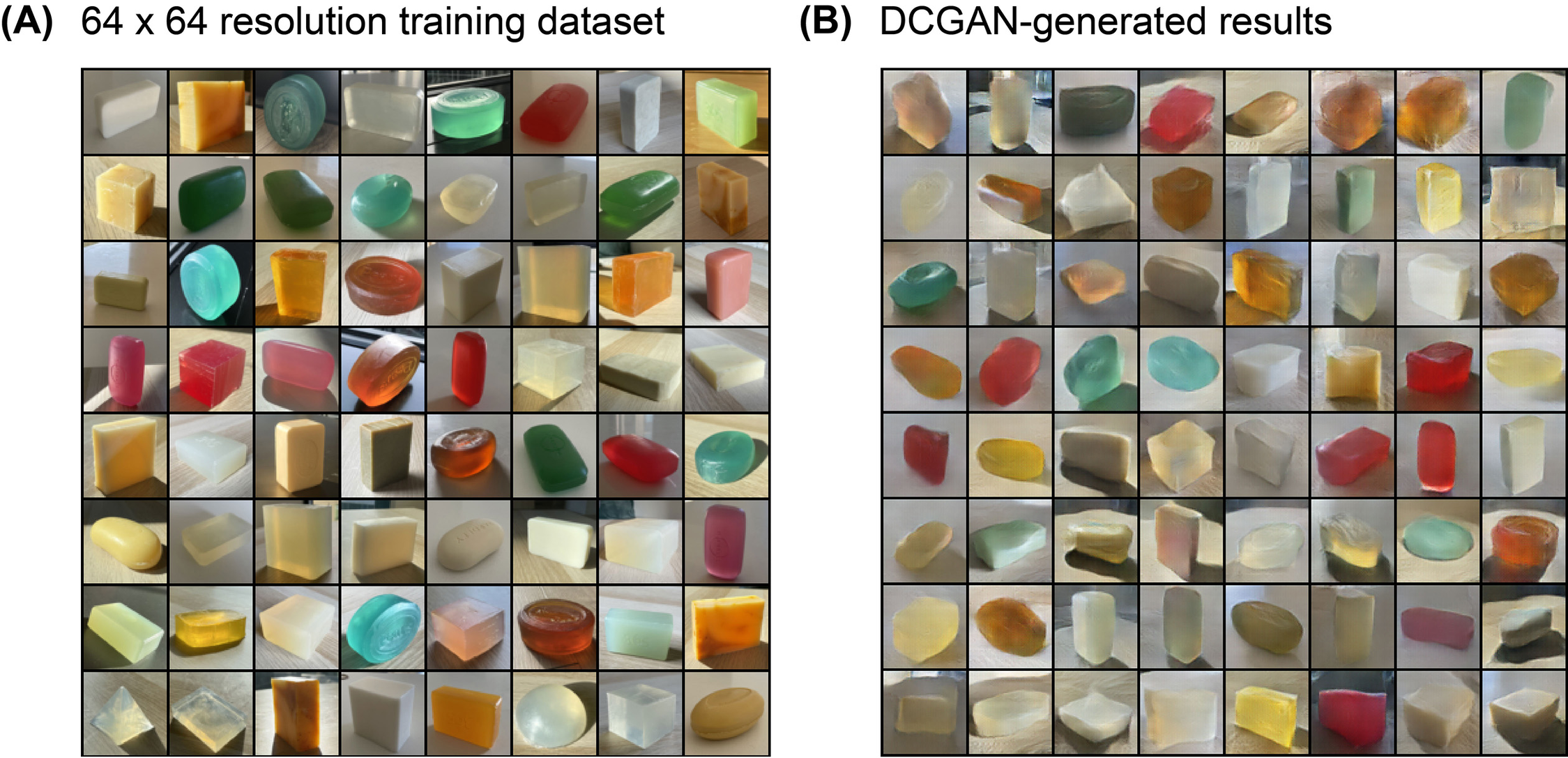

Supplement: S8 Fig — (A) Examples from the training dataset, which are images from the TID dataset, resized to 64 pixels × 64 pixels. (B) Examples of DCGAN-generated soaps after 800 epochs of training. (TIF) [file pcbi.1010878.s009.tif]

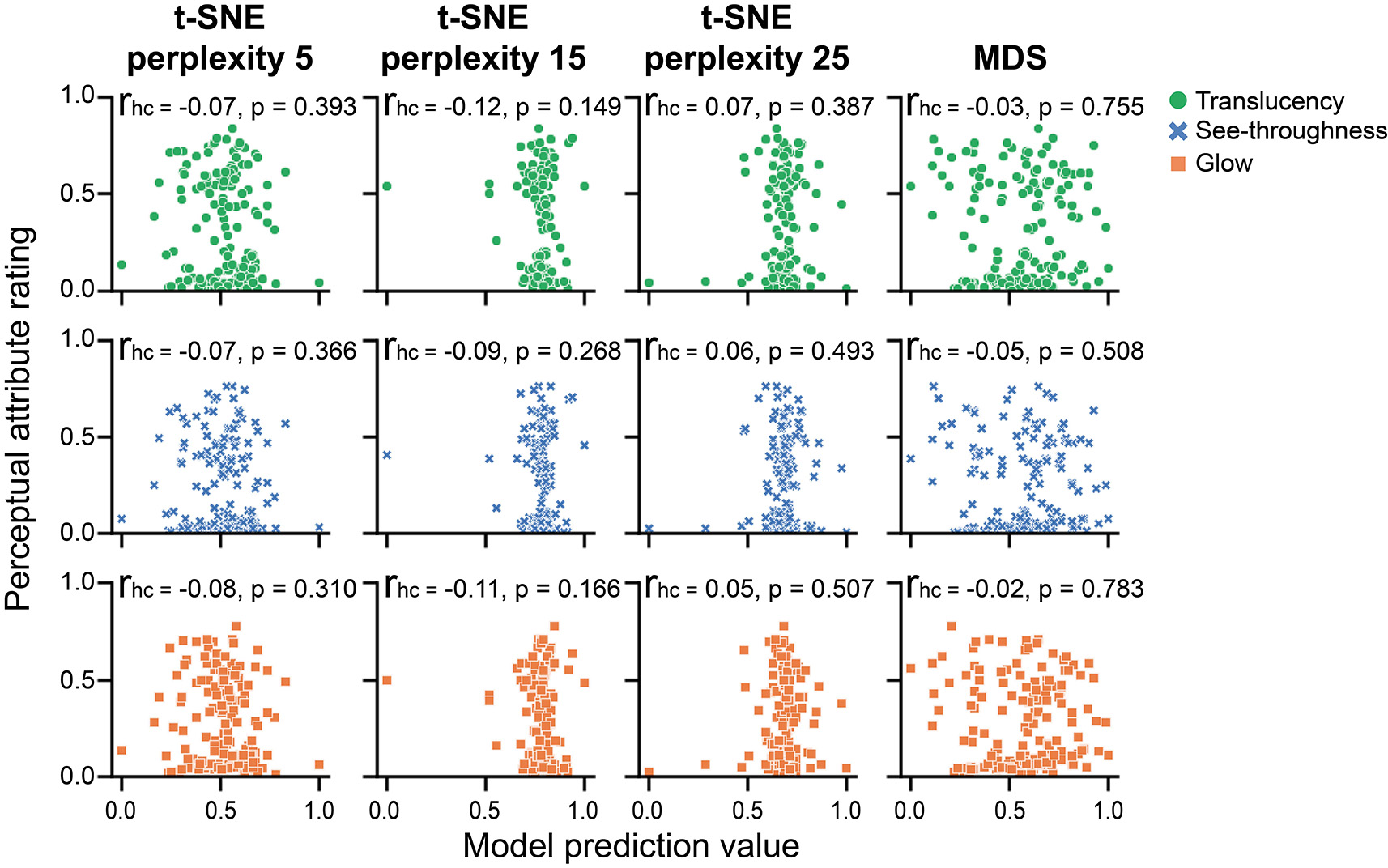

Supplement: S9 Fig — The scatter plots show the model prediction value computed from the embedding of images using dimensionality reduction methods, with the correlation coefficients (correlation between the model prediction and human perceptual ratings, rhc) and the corresponding p-values. Green, blue, and orange colors represent the data of translucency, see-throughness, and glow, respectively. (TIF) [file pcbi.1010878.s010.tif]

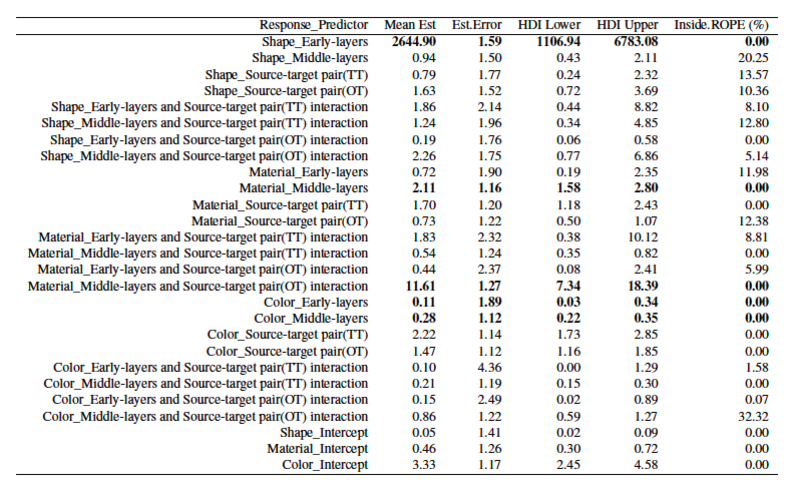

Supplement: S1 Table — The leftmost column shows the name of the parameter. The names of the response variable and the predictor are separated by “_”. The second to fifth columns are the exponentiated mean (Mean Est), the standard error (Est.Error), and the lower (HDI Lower) and upper bounds (HDI Upper) of the 95% credible interval of the posterior distribution for each parameter. The last column is the percentage of the 95% HDI of parameter distribution that falls inside the region of practical equivalence (ROPE). (TIF) [file pcbi.1010878.s011.tif]

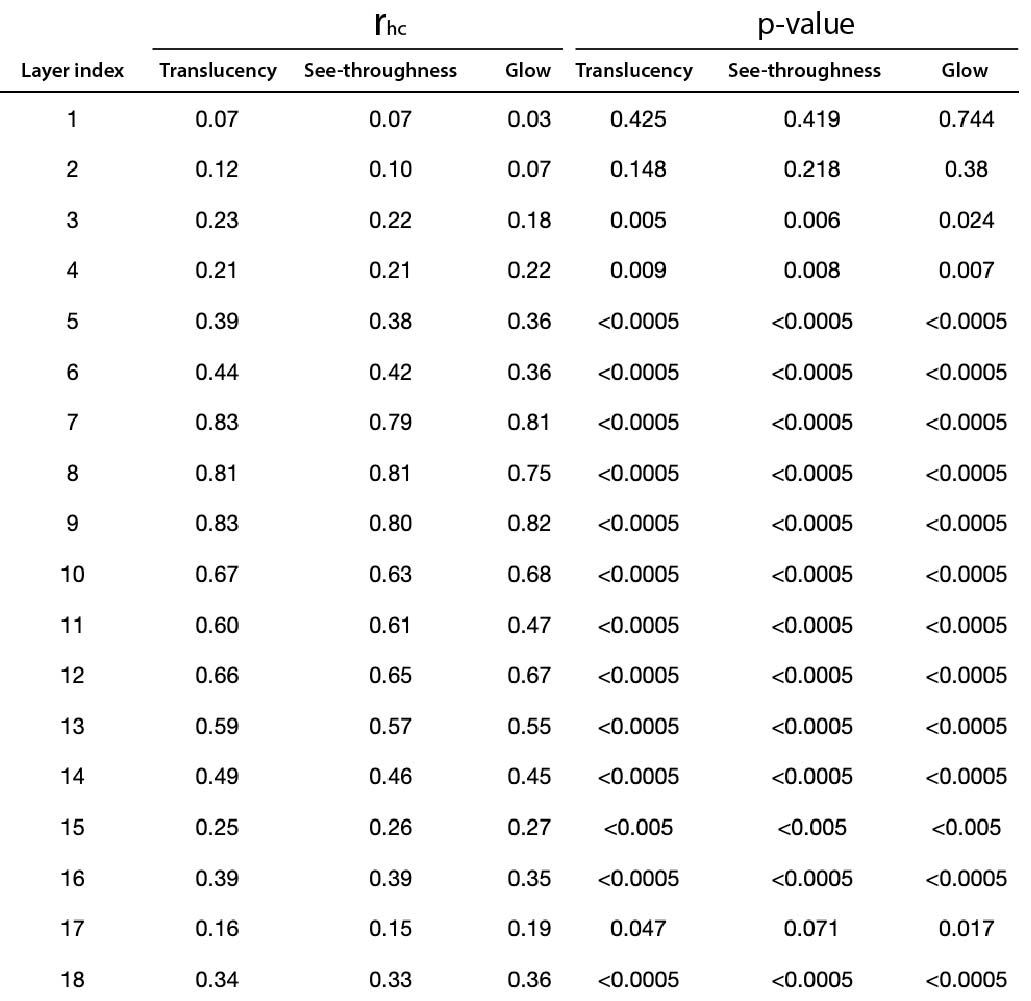

Supplement: S2 Table — The table shows the Pearson correlation between the model predictions and the mean normalized attribute ratings from Experiment 2 (columns 2 to 4), with their corresponding p-values. (TIF) [file pcbi.1010878.s012.tif]
