# Supplementary material for: Unsupervised learning reveals interpretable latent representations for translucency perception
Source: PLoS Comput Biol. 2023 Feb 8;19(2):e1010878. doi: 10.1371/journal.pcbi.1010878 (PMC9942964; doi:10.1371/journal.pcbi.1010878)
Supplement: Attachment — Submitted filename: Response_to_Reviewers.pdf [file pcbi.1010878.s013.pdf]

We thank the reviewer for the valuable feedback and think they significantly improved the manuscript. We responded point by point to the reviewer's comments below and revised our manuscript accordingly. In addition to points raised by the reviewers, we also made some changes in formatting the manuscript (see Other Changes at the end of this letter).

In the manuscript, we highlighted our changes using red-colored ink. In this letter, we use blue text as our response to the reviewers.

Reviewer #1

This paper reports unsupervised learning of translucency. Overall, I find the paper quite thought provoking and the writing to have above average clarity.

Thank you for the positive feedback.

The stimuli are rectangular prisms or ellipsoids made from various forms of soap that strongly vary in translucency. My impression is that nearly all of the example surfaces shown in the paper's figures appear to have strong back lighting. I think it is worth commenting in the main text about the distribution of illumination directions in the set of images used to train the network.

The photographs in our training dataset cover a variety of illumination directions: backlighting (44%), partial-front lighting (8%), side lighting (40%), and diffuse(dim) lighting (8%) (see Table below). The examples provided in the paper gave the impression the images mainly have backlighting conditions, which emphasize the visual phenomena of translucency (Xiao et al. 2014, Journal of Vision). In fact, the dataset contains more diverse lighting conditions. We will release the image dataset once the paper is accepted.

We added the following sentence in the Methods section:

- Line 386: "We did not intentionally/precisely balance the dataset on the distribution of shape, body color, lighting environment, and viewpoint. Approximately, our dataset covers a variety of illumination directions: backlighting (about 44%), partial-front lighting (about 8%), side lighting (about 40%), and diffuse (dim) lighting (about 8%)."

Lighting direction	Estimated proportion in dataset	Image example
Backlighting	44%	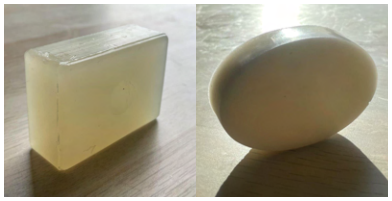

Partial-front lighting	8%	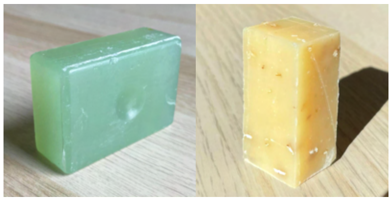
Side lighting	40%	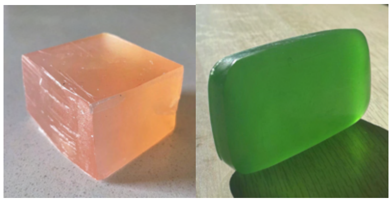
Diffuse (Dim) lighting	8%	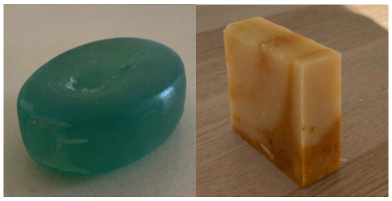

The experiments that are particularly interesting manipulate the middle layers of the network, which appears to encode information strongly related to material appearance. Specifically, the results suggest that middle layer 9 encodes information about opacity and translucency. I find that result very interesting, and it is at this point in the paper that I desired the text to provide more detail:

The basic idea is to interrogate what the network has learnt by editing the information in one layer and observing the effect on the synthesised image. Say, the network's input image is a bar of opaque soap. All of the information needed to reproduce that image is held fixed except the information in the layer whose "function" is being interrogated. The information in that layer is then replaced with the same layer's state when the input is a different surface, say a bar of translucent soap. The interesting result is that the network now synthesises an image of a surface that has the same 3D shape as the opaque soap but appears translucent. It is at this point in the text that I think it would be useful to comment on the extent of the differences between the two surfaces that are being "mixed together". How different are the overall sizes of the surfaces, their 3D shapes, colors, and illumination contexts?

The source and target can differ in size, pose, material, color, and lighting condition. Given any two images of materials (source and target, see example below), with arbitrary 3D shapes, colors, and materials, we can create a sequence of morphological transformations of visual appearance between them. We designed Experiment 3 (Figure 4 in the manuscript) to examine the semantic meaning of the layer-wise latent space. We created the stimuli (image sequences) by applying morphing on a subset of latent codes (early-layer, middle-layer, later-layer) on source-target pairs of images, conditioned only on the translucent qualities of materials (i.e.,

opaque-opaque, translucent-translucent, opaque-translucent). Other visual aspects (e.g., 3D shape, body color, lighting environment/direction, background) of the source and target images, were not conditioned and we tried to make them as diverse as possible.

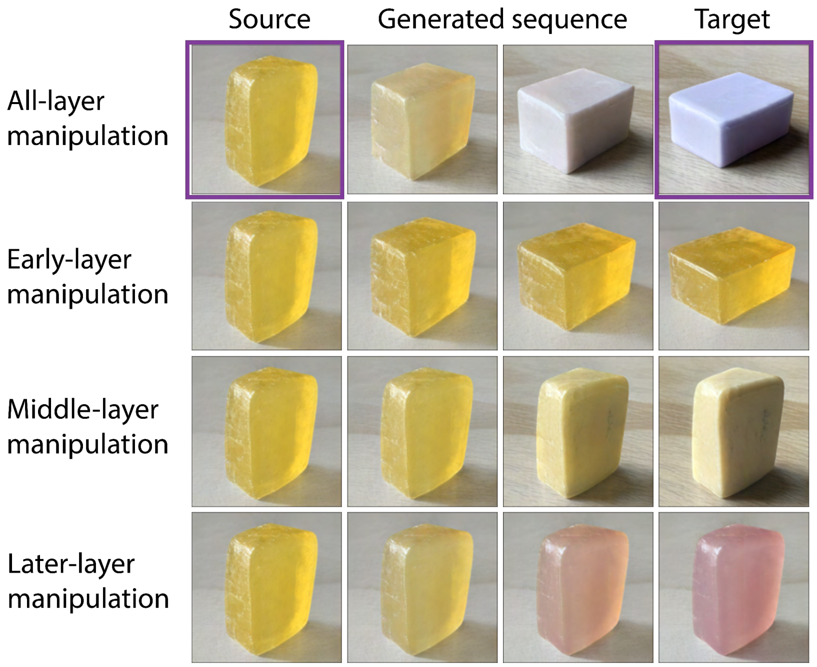

For example, in the figure above, we morph between a source (yellow glycerin soap) and a target (purple milky soap) marked by the purple boxes. The figure shows that we can create a series of intermediate images, which transform the visual appearance of the source to that of the target from all aspects (3D shape, body color, translucency, background, strengthening of lighting) by editing all layers of the layer-wise latent code. In contrast, by changing the latent codes of a subset of layers, we can coherently and independently transform certain visual characteristics between the source and target (see description in manuscript Line 162). Specifically, by changing the latent codes of middle-layers, we can modify the translucent material properties (i.e., mid-to-low spatial scale features) of the source soap while keeping the shape (i.e., low spatial scale features) invariant (see the third row in the above figure).

We edited the manuscript:

-Line 163: "Given any two generated images A (source) and B (target) with their corresponding W + latent codes w_A and w_B(512 x 18-dimensional latent codes), which could have arbitrary visual characteristics, morphing can be applied on particular layers of their latent codes to create a sequence of generated images with visual appearances lying between the source and the target."

The only other question I have is whether all the materials and code needed to reproduce this interesting result will be made available and easily accessible?

Yes. The image dataset used for model training, psychophysical experiments data, and codes will all be made available.

Data availability:

- Human psychophysics data and analysis: https://github.com/cl3789/Translucency-stylegan
- Trained models will be also shared with the public.

Reviewer #2: SUMMARY

A beautifully illustrated paper presenting a very successful technical achievement in training a generative DNN to learn the visual appearances of simple objects varying in shape, colour, and translucency. The network is trained using a custom dataset of real photographs (rather than the easier-to-collect rendered images more often used in similar work) and can generate images at an exceptionally high resolution (1024x1024 pixels). Three human behavioural experiments confirm that generated images are in many cases indistinguishable from real photographs (Expt 1), span a similar range of apparent material properties (Expt 2), and can be made to morph coherently along different appearance dimensions (Expt 3).

MAIN COMMENTS

I'm not sure how surprising is it that "human-understandable scene attributes emerge" in the latent code...this has been shown several times in several domains for GAN-based DNNs (e.g. with faces, chairs, landscapes), and is arguably necessarily true of any model that can smoothly interpolate a series of plausible objects morphing between two existing objects. Likewise with the analysis of spatial scales: it seems intuitive that small-scale layers contain information about colour (which is present within the RGB values of even small kernels), whereas coarse-scale layers specify shape (a whole-object attribute); does this tell us anything new?

In this work, we discovered a scale-specific representation of translucency, which may predict human perception. Given that many factors contribute to the perception of translucency, it is not completely intuitive that a perceptually meaningful scale-specific representation can emerge from unsupervised learning. Our model discovered a novel and plausible representation of translucency. For example, previous studies in translucency show that there are significant interactions between objects' fine geometry and material properties (Xiao et al. 2020). With our model, geometry and material properties can be relatively independently manipulated at different scales. The scale-specific representation offers a new way to separate the effects of geometry and volumetric material properties on appearance and can be used as a tool to manipulate stimuli in a systematic way while not losing their naturalness.

In addition, we believe that the emergence of scale-specific attributes is not so trivial. As in Figure 6 in the manuscript, the functional role of color kernels is a kind of white balancing, which means global image processing and is counterintuitive to high-scale computation. Although the process is applied to an overall image, it needs a white anchor, such as a white highlight. We speculate that the color attribute emerges at the final synthesizing stage of the generator because the absolute color determination needs detailed spatial content. In contrast, the relative color distribution is sufficient to compute the translucency attribute by middle-scale color kernels, though it's related to color saturation, usually defined by a white point. These findings give hypotheses for future neuroscience studies investigating hierarchical material and color processing. This discussion about color could be found in Lines 351-359.

Methodologically, with its innate ability to capture the scale-specific statistical structure of the images, StyleGAN (i.e., a generative model) can now be conceived as an apparatus that effectively simulates images of morphologically controllable materials, serving as an additional data source that supplies a compressed copy of the real image data of materials (Jahanian et al., 2022). Capturing a human-understandable scale-specific latent space of translucent materials (e.g., a variety of soaps) encourages us to envision the plausibility of exploring a wider range of materials and setting up a systematic way to examine and compare their visual representations.

We added in the Discussion section:

-Line 367: “Nevertheless, a generative model, such as StyleGAN, could be conceived as an apparatus that effectively simulates images of morphologically controllable materials, serving as an additional data source that supplies a compressed copy of the real image data of materials (Jahanian et al., 2022).”

Note: A citation is added to the Reference list “Jahanian, A., Puig, X., Tian, Y., & Isola, P. (2021). Generative models as a data source for multiview representation learning. arXiv preprint arXiv:2106.05258”.

This makes me wonder whether the authors satisfy their stated goal of "discovering perceptually relevant image features" underlying translucency perception. The network does indeed seem to have learned these features in a summary code that the authors are able to navigate; but do we end up with a clearer description of *what* these features are than we had before? Specifically, it would be wonderful to provide evidence that the representation of translucency is "human-like", rather than (just) optically accurate. e.g. perhaps by testing for quirks of human translucency perception such as the lower apparent translucency of greyscale images.

Beyond empirical observation, previous work has not systematically shown a visualization of the discovered translucency-related image features. For example, even though previous research hinted color saturation plays a role in translucency, there hasn’t been any concrete evidence of what kind of color features are relevant. Based on our middle-layer ICA analysis, we can provide a systematic and quantitative description of the visual features that are related to human

perception of translucency such as the set of oriented chromatic kennels that are descriptive of edges and glow shown in Figure 7 in the manuscript.

Additionally, the features used by the model might be “human-like”. The figure below provides image examples overlaying the data points shown in Figure 5B in the manuscript. The x-axis represents the SVM model prediction and the y-axis represents the human translucency rating reported in Experiment 2. For example, both the model and humans seem to judge the same object that is backlit to be more translucent than diffuse-lit and side-lit ones, consistent with previous human translucency work (Xiao et al., 2014, Journal of Vision). For the same oval glycerin soap (marked by pink boxes), both the model and human observers gave relatively higher translucency ratings in backlighting and side lighting conditions than the diffuse front lighting. This suggests that the image features learned by the model are perceptually relevant rather than optically accurate.

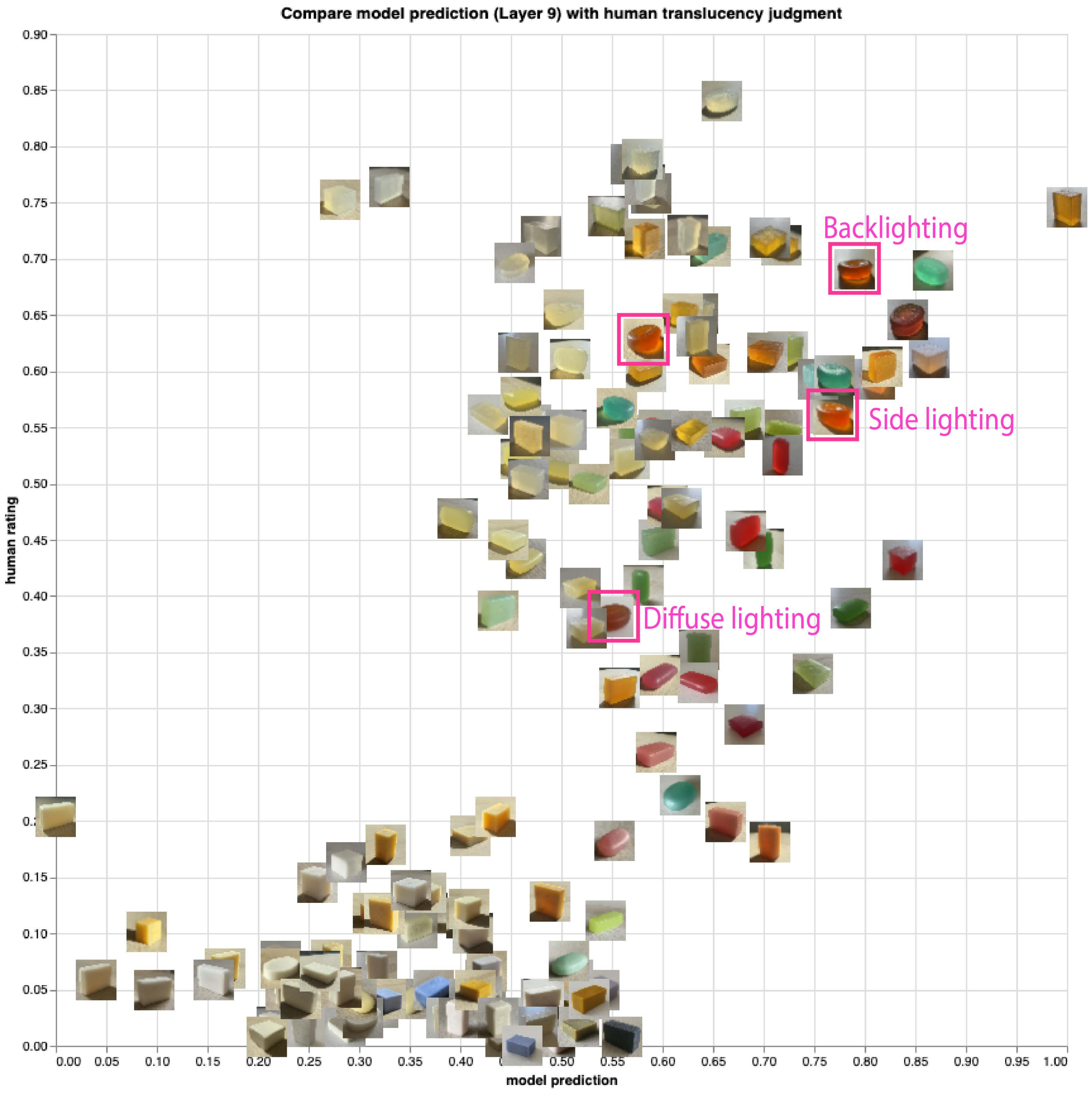

Having said that, I think the paper works as an inspiring demonstration of the potential value of carefully-curated generative networks in perception science and psychological research more broadly. The combination of network architectures (StyleGAN + a pixel-style-pixel encoder to project new images into its latent space) is well-chosen - they have been little-used in the perception science literature but solve the challenge of training on this limited dataset excellently and provide a navigable and interpretable latent space. Despite the very constrained application domain (a dataset of photographs of 60 individual soap objects), this in itself is a useful technical contribution to the field.

Thank you for the positive comments.

We preliminarily observed that our trained network can be applied to learn other translucent objects such as crystals and stones (see the images and texts included in the responses to Reviewer 3’s comments). Physically saying, refraction indices, scattering coefficients, and phase functions between soaps and stones are largely different. The success in such transfer learnings suggests that the latent factors we found capture perceptual attributes, not simply physical attributes. However, this investigation is beyond our current paper's scope, therefore, we will report them in the future.

The authors make reasonable and restrained interpretations of the results. They explicitly avoid claiming that either the architecture or learning process of the StyleGAN-based TAG network constitute good mechanistic models of human material perception. Instead they focus on the network's value as tool for data-driven discovery of complex image features that are candidates for forming the perceptual dimensions of material perception. I find this an admirably cautious and nuanced use of DNNs, and I'm sympathetic to their conclusion that "learning the scale-specific statistical structure of natural images might be crucial for...material properties".

MINOR COMMENTS

1. I'm a bit skeptical of the claim made in the Discussion (around line 307) that models without a multiscale representation (e.g. DCGAN) aren't capable of capturing translucency. Previous work shows that GANs trained on rendered images can reasonably well caputure glass-like transparency: Tamura, Prokott & Fleming (2022) https://jov.arvojournals.org/article.aspx?articleid=2778652. And "Stable Diffusion" type models currently show an outstanding ability to render the nuances of materials, including translucency - do these also have explicit multi-scale latent codes?

Yes, I agree that a simpler generative model (e.g., DCGAN) could capture some visual characteristics that roughly imply the translucent (Supplementary Fig S.9) and glassy impressions (Tamura et al., 2022). However, our results show the advantage of multiscale representations to enhance the encoding of nuanced variations of surface properties (e.g., gradual variation of saturation along surfaces/edges). In the future, we will investigate other computational models that might have different representations from StyleGAN, such as Stable Diffusion, in learning translucent material appearances.

We added the following to the Results and Discussion sections about DCGAN:

-Line 151: “Although DCGAN captured some rough visual impressions implying translucency at the relatively coarse image resolution, it is limited in synthesizing more nuanced details for perceptually convincing translucent qualities. However, the observation that the DCGAN's generative results at 64 pixels x 64 pixels resolution could already show some translucent characteristics provides empirical evidence that translucency impressions could be conveyed in a more compressed form (see Methods and Supplementary Fig S.9).”

-Line 309: “In contrast, a generative model lacking the multi-scale representational power (e.g., DCGAN) may not exquisitely represent the complex visual phenomena of translucency, despite it learns some rough visual impressions.”

2. Why ask both about "translucency" and about "see-throughness"; are these distinct concepts? The explanation given (that these were "found to be descriptive" of translucent objects in a previous study by Liao, Sawayama & Xiao (2022 JoV) doesn't fully explain it, since that study doesn't seem to have asked people to rate the two dimensions simultaneously, but rather asked for a binary translucency judgement followed by a continuous "see-throughness" judgement, and found these were closely related.

In our previous study, we find “see-thoroughness” and “translucency” were associated, but we also find they are not completely overlapping. In the Material Attribute Rating task (Experiment 2), we explained “translucency” to the observers by explicitly defining the phenomenon of subsurface scattering of light. The term “translucency” might depend more on individual interpretation while “see-throughness” is a visual term that might have a more common understanding among observers. This is because “see-throughness” provides certain explicit information about the visual quality of materials while “translucency” may include a more high-level understanding.

We added this in the Method section:

-Line 497: “While ‘see-throughness’ is a visual term that might have a more common understanding among observers, judging ‘translucency’ might depend more on individual interpretation and provide additional/complementary insights into the material qualities. Thus, both attributes were measured in this experiment.”

TYPOS etc

The paper generally reads very fluidly. There were just a few phrases that read oddly to me, e.g. from the first couple of pages:

Thank you very much for pointing them out! We adjusted them in the revised manuscript.

- line 35: "difference between raw and readily cooked food"

Correct to "difference between raw and ready-cooked food"

- line 43: "...and in the mean time, humans may lack precise descriptions"

Correct to "...and in the meantime, humans may lack precise descriptions"

- line 61: "Some recent works in perceptual system..."

Correct to "Some recent works in the perceptual system..."

Reviewer #3

This paper presents an unsupervised approach that reveals a layered representation of perceived translucency, at least for the subset of cases included in the study. It was a pleasure to read, the authors motivate their decisions convincingly enough and summarize their main findings as they move along, which always helps the reader.

I found the methodology solid, the write-up excellent, the figures well explained and informative. The perception of materials in general, and translucency in particular, is an open topic spanning different fields of research, from neuroscience to computer graphics and even robotics. I believe the paper makes a useful contribution in this regard. I found the resulting layered space intriguing, and it seems to work well.

I don't usually review nor read papers in this journal, so it may be that I'm miscalibrated, but all in all I think this is a very good paper.

I only have a few questions, comments and suggestions.

Fig. 9 in the supplemental is used by the authors to illustrate how other architectures (DCGAN) fail to generate good results. However, other than the shape, the results (for the most part) seem to actually be pretty decent regarding translucency. It'd be great if the authors could offer more insights about this.

Thank you for sharing your observations. We also addressed this in our response to Reviewer 2's minor comments. The observation from translucent images at 64 pixels x 64 pixels resolution and the corresponding DCGAN generative results might provide empirical evidence that translucency impressions could be conveyed in a more compressed form. This may support the notion that translucency impressions are established at the relatively coarse spatial scale. In everyday experience, we can usually tell whether an object is translucent or opaque from a distance. We might utilize these mid-to-low spatial scale features to quickly assess

translucency. But a more exquisite appearance is helpful in further understanding the object's material qualities. StyleGAN still has superior performance in learning the complex and intricate appearance of realistic translucent materials than DCGAN when the image resolution is much higher. We added some clarification texts about the results of DCGAN in Line 151 and Line 309.

The user study at the beginning (summarized in Fig. 2): Isn't one second too short to view the images? I wonder if the results would've varied giving the users a bit more time to analyzed the images in a more relaxed way. If possible, extending the test to maybe 5 seconds would be useful (maybe with a randomized subset of images not to make it too long)

Thank you for pointing this out. A major concern was that the increasing time of display will make the observers lose concentration on the experiment easily, and get tired as the session progresses. Even with a brief presentation of 1 second, observers might develop some strategy to detect the model-generated images (e.g., identifying some artifacts). We collected the observers' verbal feedback on what strategy they used in the Real-vs-generated Discrimination task (Experiment 1) after the experiment. Some of them mentioned that they inspected the image from the aspects that might be marginally related to the evaluation of material quality including "blurry background/edges", "how fuzzy/sharp the shadow was", "unique scratch and legibility of carved text on the surface", and "painting-like (e.g., details are not very clear)". Therefore, longer viewing time might cause the observers to pay attention to these artifacts rather than the overall material quality of the images. In Experiment 2, the observers were given unlimited time to judge the material attributes of each stimulus.

As with most papers, the results and conclusions are strictly speaking only valid for the particular subset of examples used. In that sense, and this is really my only "major" concern, some parts of the paper including the title may be a bit overstated. The authors have used only one class of translucent materials only, with basic geometries and one particular, strongly angled lighting (to emphasize the translucency effect). How do the results generalize to other setups? This would probably need to be discussed at the end of the paper, possibly showing some failure examples along with some insights as to why they may occur.

We recently examined the "plasticity" of the StyleGAN framework. Specifically, we transferred the pre-trained StyleGAN from a large soap dataset to a relatively small crystal/stone dataset, via full model fine-tuning (see figure below). With little training time, we obtained a new generator that synthesizes realistic crystals and stones with diverse material appearances. Importantly, the layer-wise latent spaces of both material domains (soap and crystal/stone) show similar scale-specific semantic representations: early-layers represent coarse spatial-scale features (e.g., the object's contour), middle-layers represent mid-level spatial-scale features (e.g., material), and later-layers represent fine spatial-scale features (e.g., color). This supports that multi-scale representation could be generalized to other materials. Even though these initial results provide a proof of concept of the transferability of our model, more work is needed to understand the full capacity of transfer learning. Thus, we will not include these results on transfer learning in the current manuscript. We discussed the limitation of using soap as a model system in the Discussion (Line 314-331).

Added in the Discussion:

-Line 327: “Constrained by the diversity in geometry and material appearance of the current training samples, our TAG model might not be directly used to synthesize images of another material that has more irregular geometries, such as crystals. However, learning about the soaps is a starting point to capture essential translucent appearances in the real world. Therefore, in future works, it is possible to extend our model to learn the representation of image data across broad categories of materials with techniques such as transfer learning.”

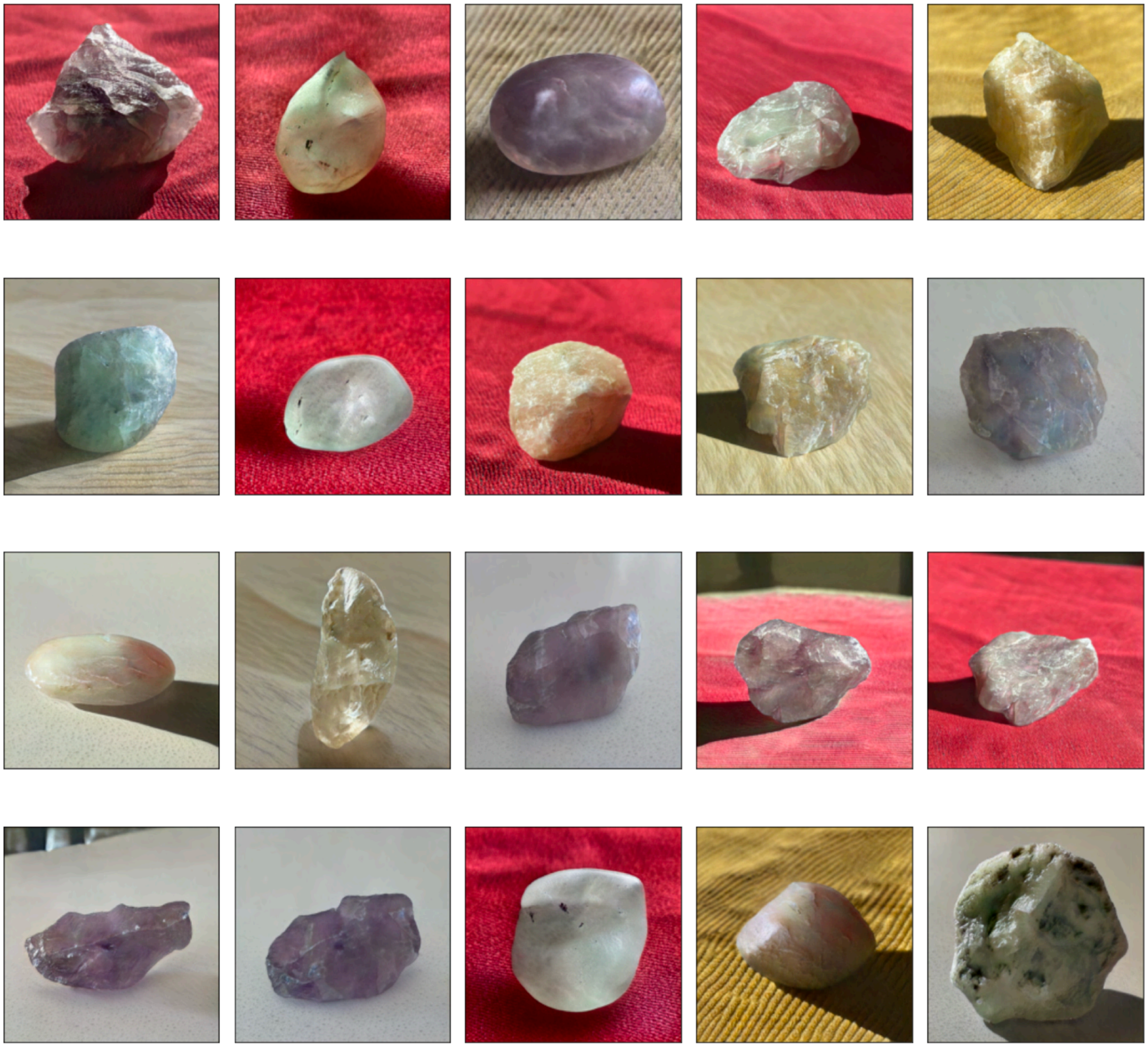

A couple of relevant papers on the topic of discovering perceptual spaces for materials could be added:

Pellacini et al. Toward a psychophysically-based light reflection model for image synthesis. SIGGRAPH 2000

Serrano et al. An intuitive control space for material appearance. ACM Transactions on Graphics, SIGGRAPH Asia 2016

Thank you. We added these citations in Line 29 and Line 52.

Note: These two citations are added to the Reference list.

Other Changes:

- The manuscript is now double-spaced.
- The texts in the Figures are changed to Arial. Within the Figures, the panels are labeled with capital letters. The reference of the figures in the text is changed from “Figure” to “Fig” (these changes are not highlighted, for better visual clarity).
